# Loss of miR-140 is a key risk factor for radiation-induced lung fibrosis through reprogramming fibroblasts and macrophages

**DOI:** 10.1038/srep39572

**Published:** 2016-12-20

**Authors:** Nadire Duru, Yongshu Zhang, Ramkishore Gernapudi, Benjamin Wolfson, Pang-Kuo Lo, Yuan Yao, Qun Zhou

**Affiliations:** 1 Department of Biochemistry and Molecular Biology, Greenebaum Cancer Center, University of Maryland School of Medicine, Baltimore, MD 21201, USA

## Abstract

Radiation-induced lung fibrosis (RILF) is a common side effect for patients with thoracic cancer receiving radiation therapy. RILF is characterized by excessive collagen deposition mediated by TGF-β1 and its downstream factor SMAD3, but the exact molecular mechanism leading to fibrosis is yet to be determined. The present study investigated the impact of miR-140 on RILF development. Herein, we first found that loss of miR-140 is a marker of fibrotic lung tissue *in vivo* one-year post-radiation treatment. We showed that miR-140 knockout primary lung fibroblasts have a higher percentage of myofibroblasts compared to wild type primary lung fibroblasts, and that loss of miR-140 expression leads to increased activation of TGF-β1 signaling as well as increased myofibroblast differentiation. We also identified fibronectin as a novel miR-140 target gene in lung fibroblasts. Finally, we have shown that miR-140 deficiency promotes accumulation of M2 macrophages in irradiated lung tissues. These data suggest that miR-140 is a key protective molecule against RILF through inhibiting myofibroblast differentiation and inflammation.

RILF is a late onset symptom of radiation-induced lung injury that usually occurs within 6 months to a year after the completion of radiation therapy^1^,^2^. RILF is characterized by scar formation due to excess wound healing and impaired lung function caused by the accumulation of macrophages, fibroblasts, myofibroblasts, and extracellular matrix (ECM) proteins^3^,^4^. Macrophage polarization plays a key role for the development and progress of RILF. Classic activation of M1 macrophages is associated with radiation pneumonitis, while alternative activation of M2 macrophages is associated with lung fibrosis. M2 macrophages cause an increased inflammatory response involving the production of inflammatory cytokines including transforming growth factor beta 1 (TGFβ1), fibroblast growth factor (FGF), platelet derived growth factor (PDGFα), and vascular endothelial growth factor (VEGF). This stimulates the growth of fibroblasts and their differentiation into myofibroblasts. Myofibroblasts are highly proliferative and contractile fibroblast-like cells that produce excessive levels of ECM proteins (collagen^5^, fibronectin^6^,^7^, elastin^8^ and fibrillin^9^) and have been shown to be crucial for the development of fibrosis^10^. Lung fibrosis permanently impairs lung function and currently has no effective treatments.

MicroRNAs (miRNAs) are short (18–24 nucleotides) endogenous non-coding RNAs that regulate a broad range of signaling pathways associated with most biological and pathological processes including cellular radiation responses and disease formation. MiRNAs bind to specific sequences in the 3′UTR of target mRNAs, resulting in mRNA degradation and translational inhibition^11^,^12^. Accumulating evidence indicates the potential role of miRNAs in the development of fibrosis in several organs including lung^13^, heart^14^,^15^,^16^, kidney^17^,^18^,^19^ and liver^20^,^21^,^22^. It has also been shown that alteration of miRNA expression is involved in radiation-induced fibrosis in a murine skin model^23^. Moreover, increased expression of miR-155 has been shown to correlate with severe lung fibrosis in bleomycin-induced mouse model^13^. Finally, it was demonstrated that low-dose paclitaxel prevents bleomycin-induced pulmonary fibrosis through the upregulation of miR-140 and subsequent suppression of the TGF-β1/Smad3 pathway^24^. While several miRNAs have been demonstrated to be involved in fibrosis, the roles of miRNAs in mechanisms of RILF are still unknown. Our lab recently identified miR-140 as a potential radio-protective molecule against radiation-induced lung injury. Using primary lung fibroblasts isolated from mice as well as human lung fibroblast cells, we have observed that radiation-induced miR-140 upregulation suppressed the sphere formation of lung fibroblasts and maintained normal fibroblast populations^25^.

In this study, we hypothesized that loss of miR-140 expression is one of the key risk factors for the development of fibrosis in lungs upon radiation treatment. Using C57BL/6 mice we demonstrated that fibrotic lung tissues have dramatically less expression of miR-140 compared to non-fibrotic lung tissues one year after radiation treatment. Treatment with a total of 20 Gy of fractionated ionizing radiation (FIR) slightly decreased the myofibroblast population of wild type mice lung fibroblasts (MLFs), while it increased the myofibroblast population of miR-140 knockout MLFs. Furthermore; we identified fibronectin as a novel target of miR-140. Finally, we showed that fibrotic lung tissues, which exhibit loss of miR-140 expression, have a dramatic increase in alternative activation of macrophages. These data suggest that miR-140 plays a critical role for RILF development. Developing drugs that upregulate miR-140 might be a novel strategy for minimizing collateral tissue damage in thoracic cancer patients being treated with IR.

## Results

### Categorization of radiation-induced fibrotic and non-fibrotic lung tissues in mice

**Figure 1:**
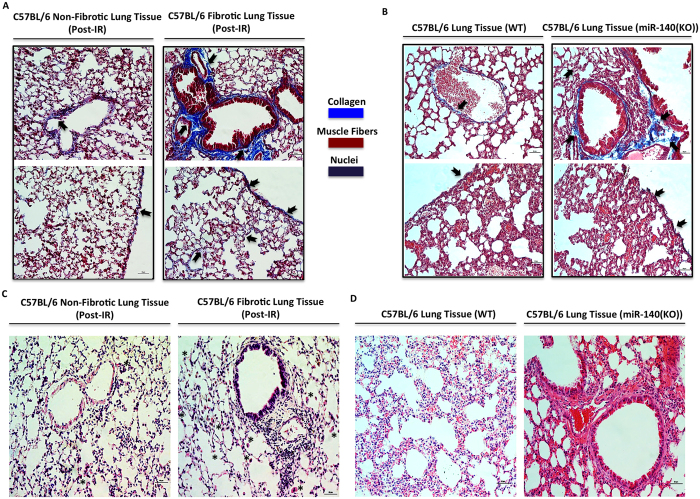
Categorization of radiation-induced fibrotic and non-fibrotic lung tissues in mice. The lungs of mice that received radiation therapy as well as the lungs of non-irradiated wild type and non-irradiated miR-140 knockout mice were assessed for the development of fibrosis using Trichrome Masson’s (blue: collagen, red: muscle fibers, black/blue: nuclei) and H&E staining. Bar scale represents 50 μm. (**A**) Trichrome Masson’s staining shows the increased collagen deposition (blue stain, black arrows) in the irradiated lung tissues with pleural effusion (n = 4) compared to the irradiated lung tissues without pleural effusion (n = 4). (**B**) Trichrome Masson’s staining showed the increased collagen deposition (blue stain, black arrows) in the lungs of non-irradiated miR-140 knockout mice compared to non-irradiated wild type mice. (**C)** H&E staining demonstrates that the irradiated lungs that have pleural effusion and increased collagen deposition are showing the morphological changes consistent with fibrotic tissues. The fibrotic lungs had thickened alveolar and bronchiolar vessels and more alveolar macrophages (asterisk) and thicker layer of airway smooth muscle. (**D)** H&E staining shows the morphological differences between non-irradiated lungs of wild type and miR-140 knockout mice. MiR-140 knockout tissue has thickened alveolar walls.

To test our hypothesis that loss of miR-140 expression plays a role in the development of RILF we used C57BL/6 mice, a common animal model for fibrosis^26^,^27^, that were irradiated in their thoracic region with 13 Gy radiation. After one year of maintenance the mice were sacrificed and their lungs were collected for further evaluation. The lungs were grouped according to the degree of pleural effusion; no effusion indicated a potentially non-fibrotic lung and high pleural effusion indicated a potentially fibrotic lung. One of the main indicators of lung fibrosis is collagen deposition. Using a modified Trichrome Masson’s staining, we measured collagen deposition in lungs. We observed a dramatic increase in collagen deposition in the lungs of mice with pleural effusions compared to those of mice with no pleural effusion 1-year post-irradiation (Fig. 1A). We also observed an increase in collagen deposition in non-irradiated miR-140 deficient lungs compared to non-irradiated wild type lungs (Fig. 1B). Higher collagen deposition in fibrotic and miR-140 deficient lungs was observed prominently in alveolar spaces at the sites surrounding bronchioles or vascular vessels. In the fibrotic lung tissues we observed fibrosis-like morphological changes including fibrous thickening of the alveolar and bronchiolar vessels and increased alveolar macrophages and enhanced thickness of airway smooth muscle layer (Fig. 1C). Furthermore, in comparison to non-irradiated miR-140 deficient lungs, non-irradiated normal lung tissues that have miR-140 expression had no inflammation (Fig. 1D). These data suggested the association of miR-140 expression with fibrotic histological changes.

### Loss of miR-140 enhances fibronectin expression in RILF

**Figure 2:**
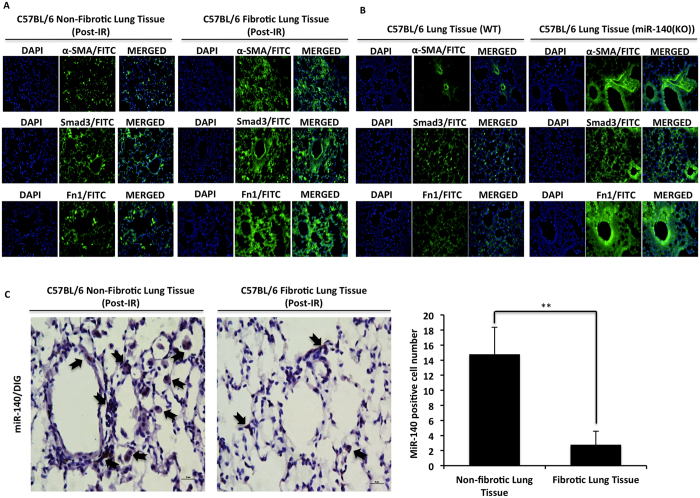
Loss of miR-140 enhances fibrosis-related protein expression in RILF. The expression levels of myofibroblast marker α-SMA, TGF-β1 signaling pathway protein Smad3 and ECM remodeling regulator protein fibronectin that are commonly overexpressed in fibrotic tissues were assessed in irradiated lung tissues with or without pleural effusion as well as non-irradiated lung tissues from wild type and miR-140 knockout mice. (**A)** Immunofluorescent staining of fibrotic lung tissues shows the increased expression of α-SMA, Smad3 and fibronectin compared to non-fibrotic lung tissues. Quantification of the data is shown in Supplementary Figure S1A. (**B)** Immunofluorescent staining showing the increased α-SMA, Smad3 and fibronectin expression in non-irradiated miR-140 knockout lung tissue compared to non-irradiated wild type lung tissue. Quantification of the data is shown in Supplementary Figure S1B. (**C**) *In situ* hybridization shows that non-fibrotic lung tissues have significantly higher number of miR-140 positive cells compared to fibrotic tissues 1 year after irradiation. Bar scale represents 50 μm. Data represent the mean ± S.D. (n = 3). **p < 0.01.

The TGF-β1 signaling pathway is one of the key pathways in fibrogenesis and mediates the excessive deposition of collagen and fibronectin through its downstream signaling pathway protein Smad3^28^,^29^. To further understand the impact of miR-140 expression on *in vivo* fibrosis development, we used immunofluorescence staining to assess the expression of myofibroblast marker α-SMA, TGF-β1 signaling activator Smad3, and ECM remodeling regulator protein fibronectin in lung tissues 1 year post-irradiation. The expression of α-SMA, Smad3, and fibronectin were significantly higher in fibrotic lung tissues compared to non-fibrotic lung tissues (Fig. 2A). Moreover, the expression of α-SMA, Smad3, and fibronectin was also significantly higher in non-irradiated miR-140 knockout lung tissue compared to non-irradiated wild type lung tissue (Fig. 2B). Finally, to examine the expression of miR-140 in non-fibrotic and fibrotic lung tissues, we performed *in-situ* hybridization staining using a miR-140 RNA probe labeled with 5′-digoxigenin and found that non-fibrotic lung tissue had a significantly higher number of miR-140-positive cells compared to fibrotic lung tissues 1 year after radiation treatment (Fig. 2C). These results suggest that miR-140 protects the lung tissue from RILF through inhibiting α-SMA, Smad3, and fibronectin expression.

### Loss of miR-140 leads to increased myofibroblast formation in mice lung fibroblasts

**Figure 3:**
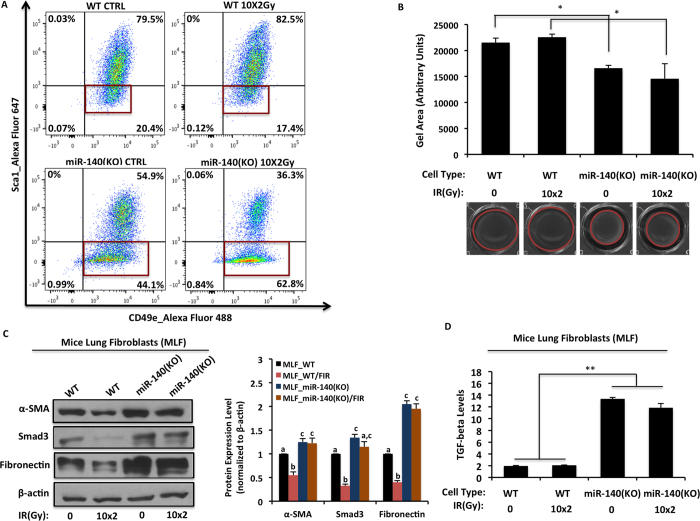
Loss of miR-140 leads to increased myofibroblast formation in lung fibroblasts. Non-irradiated and fractionated irradiated (10x2Gy) wild type MLFs and miR-140 knockout MLFs were harvested 24 h after the last treatment and myofibroblast formation was measured using FACS, collagen contraction, western blot and ELISA assays. (**A)** Myofibroblast formation was assessed using Sca-1 and CD49e markers via FACS analysis. MiR-140 knockout MLFs had more myofibroblast population (Sca-1^low^/CD49e^high^) compared to wild type MLFs in the absence of radiation treatment. Fractionated irradiation induced the myofibroblast formation in miR-140 knockout MLFs and decreased in wild type MLFs. (**B**) Evaluation of the impact of myofibroblast formation in MLFs with collagen gel contraction assays. MiR-140 knockout MLFs contracted significantly more compared to wild type MLFs indicating higher myofibroblast content. FIR treatment slightly reduced the contraction in wild type MLFs while it slightly increased in the miR-140 knockout MLFs. The contraction capacity was calculated by measuring the gel area with ImageJ 24 h after the stress was lifted. Data represent the mean ± S.D. (n = 3). *p < 0.05. (**C)** The expression levels of α-SMA, Smad3 and fibronectin were analyzed using western blot. MiR-140 knockout MLFs had a higher expression of α-SMA, Smad3 and fibronectin compared to wild type MLFs. Fractionated radiation induced the expression of α-SMA, Smad3 and fibronectin in miR-140 knockout MLFs while a reduction of these proteins were observed in wild type MLFs. β-actin was used as loading control. In the figure are reported the cropped gels/blots obtained by each protein evaluation. All gels were run in the same experimental conditions. Full-length blots of each tested protein are reported in Supplementary Figure S2. (**D)** The level of TGF-β1, one of the key elements of fibrogenesis was assed using ELISA assay. MiR-140 knockout MLFs had more TGF-β1 levels compared to wild type MLFs. Data represent the mean ± S.D. (n = 3). **p < 0.01.

Myofibroblast differentiation is one of the hallmarks of fibrosis. Myofibroblasts express high levels of α-SMA^30^, and can be isolated using the markers Sca-1^low^/CD49e^high ^31. Since we found a dramatic decrease of miR-140 expression in fibrotic tissues, we hypothesized that miR-140 might have a role in regulating myofibroblast formation in lung fibroblasts. We isolated lung fibroblasts from wild type and miR-140 knockout mice and delivered FIR with a total dose of 20 Gy over two weeks (10x2Gy) to mimic the clinical treatment schedule for cancer patients. We performed fluorescence-activated cell sorting (FACS) and isolated the myofibroblast subpopulations within the mouse lung fibroblast (MLFs) population. Our results show that miR-140 knockout MLFs contained a significantly higher Sca-1^low^/CD49e^high^ myofibroblast population (44.1%) compared to wild type MLFs (20.4%). Interestingly, upon FIR treatment the Sca-1^low^/CD49e^high^ subpopulation from wild type MLFs dropped to 17.4%, whereas the Sca-1^low^/CD49e^high^ subpopulation from miR-140 knockout MLFs increased almost 20% (62.8%) (Fig. 3A). These data suggest that loss of miR-140 expression promotes FIR-induced myofibroblast formation. We then examined the impact of the increased myofibroblast population on cell contraction using a collagen contraction assay. We observed that miR-140 knockout MLFs have the ability to contract more compared to wild type MLFs (Fig. 3B). Next, we compared the protein expression levels of α-SMA, Smad3 and fibronectin in wild type and miR-140 knockout MLFs (Fig. 3C) and found that all three proteins were upregulated in miR-140 knockout MLFs compared to wild type MLFs. Interestingly, while we saw a dramatic decrease in α-SMA, Smad3 and fibronectin expression in wild type MLFs 24 hours after FIR treatment, we did not observe any decrease in the expression of α-SMA, Smad3 or fibronectin in miR-140 knockout cells. This result shows that miR-140 expression is necessary for radiation-induced suppression of α-SMA, Smad3 and fibronectin. The overexpression of Smad3 and fibronectin in miR-140 knockout MLFs suggested that TGF-β1 signaling is activated in miR-140 knockout MLFs. To confirm this, we performed an ELISA assay to measure the TGF-β1 levels from the supernatants of irradiated or non-irradiated MLFs. TGF-β1 levels were significantly higher in miR-140 knockout MLFs compared to wild type MLFs (Fig. 3D). Moreover, irradiated miR-140 knockout MLFs also had higher TGF-β1 levels compared to irradiated wild type MLFs. These data suggest that loss of miR-140 causes the activation of TGF-β1 signaling and enhances fibronectin expression.

### MiR-140 inhibits fibronectin in lung fibroblasts

**Figure 4:**
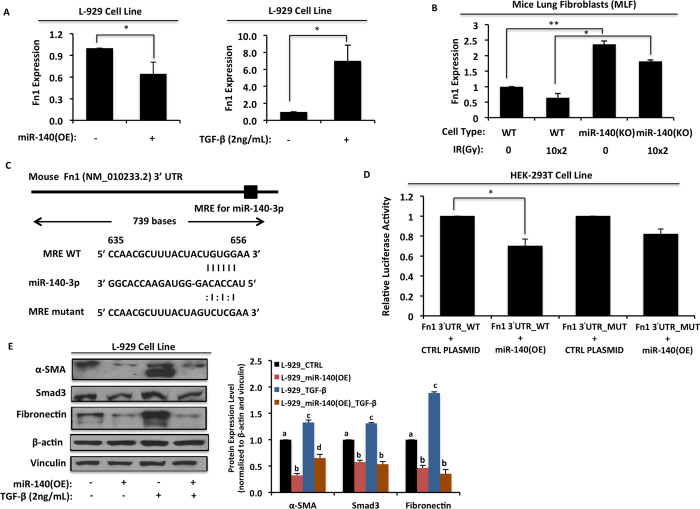
MiR-140 inhibits fibronectin in lung fibroblasts. **(A)** L-929 mice fibroblasts transfected with miR-140 overexpressing plasmid or treated with TGF-β (2 ng/ml) and the expression level of fibronectin was measured with RT-PCR. MiR-140 overexpression significantly decreased the fibronectin expression in contrast to TGF-β treatment, which significantly increased the fibronectin expression. Data represent the mean ± S.D. (n = 3). *p < 0.05. **(B)** Non-irradiated and FIR treated (10x2Gy) MLFs were harvested 24 h after the last treatment and the expression level of fibronectin was assed via RT-PCR. MiR-140 knockout MLFs cells had a significantly higher level of fibronectin expression compared to wild type MLFs. Data represent the mean ± S.D. (n = 3). *p < 0.05, **p < 0.01. **(C)** The miR-140 response element in fibronectin 3′-UTR as predicted by microRNA.org and mutated bases of the predicted miR-140 target seeding site. (**D)** Transfection of HEK-293T cells decreased the wild type fibronectin 3′-UTR reporter activity by 30% compared to transfection of empty vector controls. Transfection of miR-140 expression vector did not significantly alter mutant fibronectin 3′-UTR reporter activity. Data represent the mean ± S.D. (n = 3). *p < 0.05. (**E)** The expression levels of α-SMA, Smad3 and fibronectin in L-929 mouse fibroblasts transfected with or without miR-140 overexpressing plasmid and treated with or without 2 ng/mL TGF-β were analyzed using western blot. MiR-140 overexpression caused a dramatic decrease in the expression of all three proteins and aborted the effect of TGF-β treatment. In the figure are reported the cropped gels/blots obtained by each protein evaluation. All gels were run in the same experimental conditions. Full-length blots of each tested protein are reported in Supplementary Figure S3.

Fibronectin is an ECM remodeling regulator protein associated with collagen deposition in lung tissues. Our western blot data showed that miR-140 knockout MLFs express significantly higher levels of fibronectin compared to wild type MLFs. We hypothesized that miR-140 could exert its anti-fibrotic effect through directly targeting the 3′UTR of *fibronectin* mRNA. To test if *fibronectin* is a novel target of miR-140, we overexpressed miR-140 in L-929 mouse fibroblasts and measured *fibronectin* mRNA expression using qRT-PCR. Overexpressing miR-140 significantly decreased *fibronectin* mRNA expression (Fig. 4A, Left Panel). Using the same cell line, we also confirmed that TGF-β1 treatment causes a dramatic increase in *fibronectin* mRNA expression levels (Fig. 4A, Right Panel). Furthermore, miR-140 knockout MLFs had significantly higher fibronectin expression compared to wild type MLFs (Fig. 4B). Based on these findings, we performed *in silico* analysis to identify a potential mechanism for fibronectin downregulation. We found that miR-140 is predicted to target a conserved site within the fibronectin 3′-UTR (Fig. 4C). To test whether miR-140 could regulate the predicted target site within the fibronectin mRNA 3′-UTR, we cloned the wild type fibronectin 3′-UTR luciferase reporters. We also constructed mutant luciferase reporters by mutating 3 bases of the predicted miR-140 target seeding site (TACTA(C to G)T(G to C)T(G to C)GAAAGACAA). HEK-293T cells were co-transfected with wild type or mutant pSGG miR-140 3′-UTR luciferase vectors (along with phGR-TK *Renilla* luciferase vectors for normalization) in addition to miR-140 expression vector or empty vector control. Co-transfection of miR-140 expression vector was found to decrease wild type fibronectin 3′-UTR reporter activity by 30% compared with transfection of empty vector controls. However, co-transfection of miR-140 expression vector did not significantly alter mutant fibronectin 3′-UTR reporter activity (Fig. 4D), suggesting that miR-140 targets the 3′-UTR of *fibronectin* mRNA. To test the impact of miR-140 on protein levels of fibronectin in L-929 mouse fibroblasts, the cells were transfected with miR-140 overexpressing vector or empty vector. We treated these cells with or without TGF-β1 (2ng/mL). Treatment with TGF-β1 enhanced expression levels of α-SMA, Smad3 and fibronectin. However miR-140 overexpression inhibited TGF-β1-induced protein levels of α-SMA, Smad3 and fibronectin (Fig. 4E), suggesting that fibronectin is a novel target of miR-140 and that miR-140 inhibits TGF-β1 signaling in lung fibroblasts.

### M2 macrophages are enhanced in miR-140 deficient lung tissue and fibroblasts

**Figure 5:**
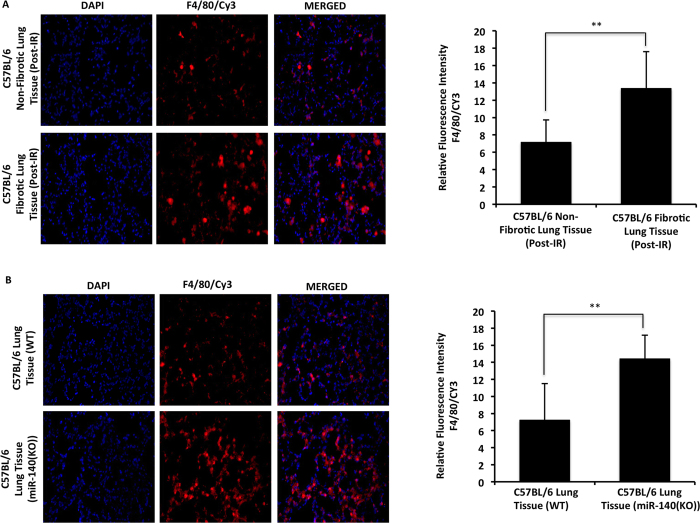
Increased macrophage number in miR-140 deficient and fibrotic lung tissues. Pan-macrophage marker F4/80 was assessed in irradiated fibrotic and non-fibrotic, as well as non-irradiated wild type and MiR-140 knockout lung tissues. (**A)** Immunofluorescent staining shows that fibrotic lung tissues have significantly higher number of F4/80 positive macrophages compared to non-fibrotic lung tissues one year after radiation treatment and (**B)** MiR-140 knockout lung tissues have higher number of F4/80 positive macrophages compared to wild type lung tissues in the absence of radiation treatment.

**Figure 6:**
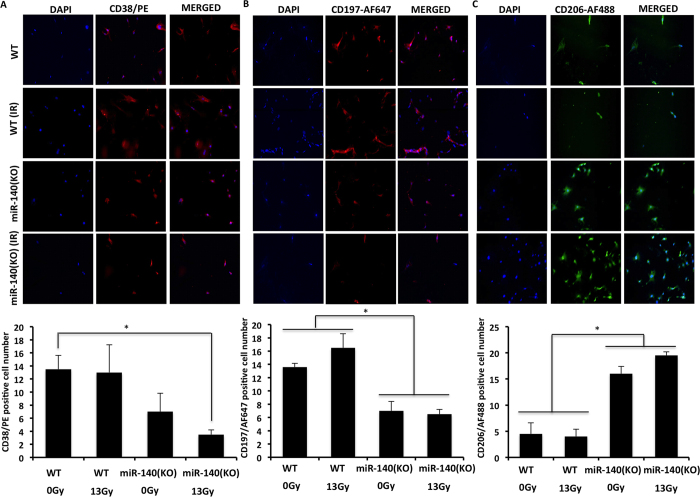
Alternative activation of macrophages is enhanced in miR-140 knockout MLFs. Markers associated with M1 macrophages (CD197, CD38) and M2 macrophages (CD206) were assessed in wild type MLFs and miR-40 knockout MLFs. Wild type MLFs and miR-140 knockout MLFs were treated with or without 13 Gy radiation and assessed for the expression of markers associated with M1 macrophages CD38 **(A)** and CD197 **(B)** as well as M2 macrophages CD206 **(C)**.

**Figure 7:**
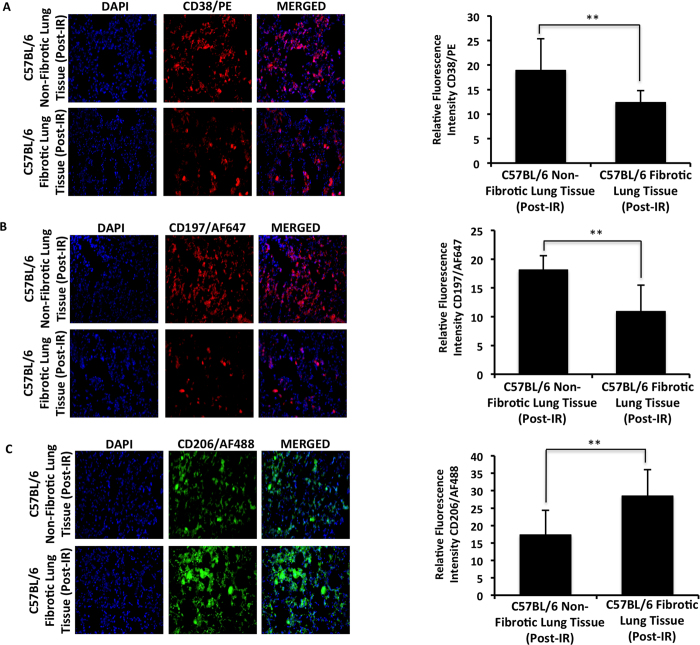
Alternative activation of macrophages is enhanced in fibrotic lung tissue. Markers associated with M1 macrophages (CD197, CD38) and M2 macrophages (CD206) were assessed in fibrotic and non-fibrotic lung tissues. (**A,B)** Immunofluorescent staining of fibrotic lung tissues shows significantly decreased expression of CD38 **(A)** and CD197 **(B)** compared to non-fibrotic lung tissues one year after radiation treatment. (**C)** CD206 is expressed significantly more in fibrotic tissues compared to non-fibrotic tissues one year after radiation treatment.

Lung fibrosis is associated with an increased number of F4/80+ macrophages^32^,^33^. Our H&E staining indicated that fibrotic lungs had a higher number of alveolar macrophages compared to non-fibrotic lungs. We confirmed this by showing the increased number of F4/80+ macrophage cells in fibrotic lungs compared to non-fibrotic lungs (Fig. 5A). Furthermore, miR-140 knockout lung tissue had significantly increased F4/80+ macrophages compared to wild type lung tissue (Fig. 5B). While the F4/80+ staining indicates a global increase in macrophages in fibrotic lungs, alternatively activated M2 macrophages have been shown to play a role in fibrosis. M2 macrophages increase the TGF-β levels leading to myofibroblast differentiation and eventually promoting fibrosis^34^. Thus, we wanted to investigate the impact of miR-140 expression on macrophage polarization. We treated wild type MLFs and miR-140 knockout MLFs with 13 Gy radiation and assessed the impact of radiation treatment on macrophage phenotypes. 24-hours post-radiation the cells were stained with M1 macrophage markers CD38 and CD197 or M2 macrophage marker CD206. In the absence of radiation, wild type MLFs had higher expression of CD38 and CD197 compared to miR-140 knockout MLFs (Fig. 6A,B). M2 marker CD206, on the other hand, was expressed significantly higher in miR-140 knockout MLFs compared to wild type MLFs (Fig. 6C). Treatment of miR-140 knockout MLFs with 13 Gy radiation slightly decreased the expression of CD38 and CD197 compared to non-irradiated miR-140 knockout MLFs. Moreover, irradiated miR-140 knockout MLFs had significantly higher expression of CD206 compared to non-irradiated miR-140 knockout MLFs. These data suggested that miR-140 deficiency leads to enhanced expression of M2 macrophages. To investigate the potential impact of miR-140 expression on macrophage phenotype *in vivo*, we performed immunofluorescent staining on fibrotic and non-fibrotic lung tissues using CD38, CD197, and CD206 markers. The results showed that the expression of M1 markers CD38 and CD197 were dramatically higher in non-fibrotic tissues compared to fibrotic tissues (Fig. 7A,B). However, the expression of M2 marker CD206 was significantly increased in the fibrotic tissues compared to non-fibrotic tissues (Fig. 7C). These data support that miR-140 is a key molecule in the regulation of macrophage polarization.

## Discussion

The TGF-β1signaling pathway is one of the key pathways responsible for lung fibrosis^35^. In the present study, we have identified that loss of miR-140 is a key risk factor for RILF. Therapeutic activation of miR-140 may have protective roles against RILF development through the suppression of myofibroblast differentiation, degradation of fibronectin, and inhibition of alternative activation of macrophages.

Our previous studies demonstrated the radioprotective effects of miR-140 expression in human lung fibroblasts^25^. In our current study we hypothesized that loss of miR-140 might be a key risk factor for RILF development. We found a dramatic decrease in the expression of miR-140 in fibrotic lung tissues compared to non-fibrotic lung tissues one-year post-radiation treatment using the C57BL/6 mice irradiated with 13 Gy radiation. To elucidate the mechanism of miR-140 in regulation of RILF we used wild type and miR-140 knockout primary MLFs and investigated whether miR-140 impacts myofibroblast formation. Using Sca1/CD49e murine myofibroblast markers we showed that myofibroblasts are significantly enriched in miR-140 knockout MLFs compared to wild type MLFs. While FIR treatment caused a significant increase in myofibroblast differentiation from miR-140 knockout MLFs, it decreased the myofibroblast differentiation of wild type MLFs. This suggests that radiation-induced miR-140 expression inhibited the formation of myofibroblasts. We also found that TGF-β1 signaling, myofibroblast marker α-SMA and ECM remodeling regulator protein fibronectin were expressed at higher levels in miR-140 knockout MLFs compared to wild type MLFs. Our data demonstrate that loss of miR-140 leads to increased fibronectin expression, which is critical for RILF development. We found that miR-140 directly binds to the 3′UTR of fibronectin and that miR-140 overexpression causes a decrease in fibronectin expression. This finding suggests that fibronectin is a novel direct target of miR-140. MiR-140 has previously been shown to target SMAD3^36^. However, TGF-β1-induced fibronectin synthesis was independent of Smad2 or Smad3 expression^37^. Our findings suggest that miR-140 maintains normal ECM remodeling through inhibition of fibronectin expression.

The classical activation of macrophages has been associated with radiation-induced pneumonitis, and alternative activation of macrophages has been associated with RILF^38^,^39^. However, the mechanism underlying macrophage polarization in RIFL is not fully elucidated. We have found that lung tissues that became fibrotic one-year after radiation treatment had significantly lower expression of M1 macrophage markers CD38 and CD197 and significantly higher expression of M2 marker CD206 compared to non-fibrotic lung tissues. Reports demonstrate that M2 macrophages are the prominent macrophage type in patients with pulmonary fibrosis while pro-inflammatory M1 macrophages are shown to protect mice from developing pulmonary fibrosis^40^. Furthermore, M2 macrophages secrete high levels of TGF-β1 and contribute to collagen synthesis^41^. The balanced transition from pro-inflammatory M1 macrophages to pro-fibrotic and wound healing M2 macrophages is crucial for the normal healing of tissue after injury. It is possible that after radiation-treatment miR-140 plays a key role in maintaining this balance by regulating the macrophage polarization towards the M1 macrophages and antagonizing TGF-β1 activation, which promotes normal tissue repair and prevents the development of fibrosis. In the absence of miR-140, M2 macrophages prevail and with constant wound healing of the lung tissue scar tissue accumulates and contributes to the development of fibrosis. Overall, our data suggest that loss of miR-140 contributes to activation of M2 macrophages in RILF. Previous studies showed that knocking down Smad3 provides protection against macrophage infiltration in pulmonary fibrosis^42^. It is likely that miR-140 expression is required to suppress TGF-β1-promoted polarization of M2 macrophages. Our future studies will characterize mechanisms of miR-140 in regulation of M1 and M2 macrophages.

In summary, our data indicate that loss of miR-140 expression contributes to RILF development. We show that miR-140 exerts its anti-fibrotic effects through multiple mechanisms including inhibition of myofibroblast differentiation, the TGF-β1/Smad3 signaling pathway, fibronectin, and the alternative activation of macrophages. Further studies will reveal if upregulation of miR-140 in concurrence with radiotherapy is an effective therapeutic strategy against RILF.

## Materials and Methods

All experiments were performed in accordance with approved guidelines and regulations set by University of Maryland School of Medicine. The Animal Care and Use Committee at the University of Maryland School of Medicine approved the care and use of all mice.

### Cell culture and Reagents

Mice fibroblast cells (L-929) were purchased from American Type Culture Collection (ATCC). They were cultured in Eagle’s Minimum Essential Medium (EMEM) (ATCC, Manassas, VA) supplemented with 10% heat-inactivated horse serum (HS) (Invitrogen, Carlsbad, CA). HEK-293T cells were obtained from ATCC and were grown in Dulbecco’s modified Eagle’s medium (DMEM) with 5% heat-inactivated fetal bovine serum (FBS) (HyClone; Rockford, IL) and 1% L-glutamine. The cells were incubated in 5% CO_2_ at 37 °C. Mouse recombinant TGF‐β1 was purchased from Cell Signaling technology (Denvers, MA).

### Isolation and Culture of Mice Lung Fibroblast

The protocol to isolate fibroblast cells from the mice lungs was adapted from Seluanov *et al*.^43^. The Animal Care and Use Committee at the University of Maryland School of Medicine approved the care and use of all mice. Briefly, wild type and miR-140 knockout C57BL/6 mice were sacrificed and their lungs were harvested. Small pieces of lungs were incubated in dissociation buffer containing 10X Collagenase/Hyaluronidase and P/S. The tissue pieces were washed with PBS and were incubated in the attachment/growth medium (DMEM, 1% P/S, 15% FBS). Once cells attached and started to grow the medium was changed to EMEM to support the growth of fibroblasts.

### Irradiation of the Cells and Mice

Radiation was delivered to cultured cells using a Gamacell 3000 gamma irradiator (dose rate: 3.75 Gy/min; Nordion International Inc). C57BL/6 mice were irradiated with 13 Gy single dose and their lungs were harvested 1 year post-irradiation. These lung tissues are kindly provided by Dr. Isabel L. Jackson (Department of Radiation Oncology at University of Maryland School of Medicine).

### FACS analysis of surface antigen proteins

Fluorescence-activated cell sorting (FACS) analysis was performed using a FACSAria II cell sorter (Beckman Coulter, Fullerton, CA, USA). Cells were blocked with anti-mouse FcR antibody (CD16/CD32) (BD Biosciences, San Jose, CA) for 15 min at 4 °C in FACS buffer (PBS with 2% FBS and 1 mM EDTA). Cells were then stained with the surface antibodies for 20 min on ice. After they were washed twice in the FACS buffer they were resuspended and analyzed immediately. The following antibodies from Biolegend (San Diego, CA) were used to identify the myofibroblast population: Sac-1/Alexa Fluor 647 and CD49e/Alexa Fluor 488.

### Western Blotting

Total cell lysates (10–30 μg) were separated by SDS-PAGE and blotted onto polyvinylidene difluoride membrane. The membrane was incubated with specific primary antibody overnight followed by the horseradish peroxidase (HRP)-conjugated secondary antibody, and visualized by the ECL Western blotting detection system (Thermo Scientific; Rockford, IL). β-actin and vinculin were both purchased from Sigma (St Louis, MO) and were used as the loading control. Antibodies against α-SMA, SMAD3 and Fibronectin were purchased from Dako (Carpinteria, CA), Santa Cruz (Dallas, TX) and Millipore (Billerica, MA).

### Enzyme-Linked Immunosorbent Assay (ELISA) for TGF-β

TGF-β protein levels of the supernatants of the cell cultures of the interest were measured using the enzyme-linked immunosorbent assay (ELISA) kit (eBioscience, San Diego, CA) following the manufacturers’ protocols.

### Collagen Contraction Assay

Collagen gel contraction assay was set up according to manufacturer’s instructions (Cell Biolabs; CBA-201; San Diego, CA). In brief, a cell suspension of 2.0 × 10^6^ cells/ml was obtained. 100 μl of the cell suspension was mixed with 400 μl of neutralized collagen solution and added to one well of a 24-well cell culture plate and allowed to solidify for one hour at 37 °C. After polymerization, 1 ml of complete media was added to each well and the cells were incubated for 48 h. The stress was released by running a sterile pipette tip along the sides of the well. The culture dish was then scanned immediately after the stress was released (time 0) and at the other time points that was determined. The area of the collagen gel was then measured using ImageJ software (NIH).

### Quantitative real-time PCR (qRT-PCR)

Total RNA was extracted with TRIzol reagent (Invitrogen; Carlsbad, CA) and analysis of mRNA expression was performed as described previously with normalization to GAPDH^44^.

### Plasmid Constructs and Luciferase Assay

The Fn1 3′-UTR was amplified by PCR using the primers 5′-ACGTTTGCTAGCATCTTTCCAGCCCCACCCTA-3′ and 5′-ACGTTT GTCGACgacaatttttCTCTCCAAACACA-3′ and cloned into the NheI and XhoI sites of pSGG vector. The plasmid obtained was then used as template to generate a mutant miR response element for miR-140-3p using the Generate site-directed mutagenesis system (Invitrogen, Carlsbad, CA) and primers 5′-tctttttattaaaacacttgtctttcgagactagtaaagcgttggcatgtgcttat-3′ and 5′-ataagcacatgccaacgctttactagtctcgaaagacaagtgttttaataaaaaga-3′. The mutant contained three point mutations, tacta(c to g)t(g to c)t(g to c)gaaagacaa, as confirmed by sequencing. HEK293T cells were seeded in twelve-well plates (3 × 10^5^/well) and transfected with pGL3 luciferase vector containing wild type fibronectin 3′-UTR or mutant fibronectin 3′-UTR by Lipofectamine 2000 (Invitrogen, Carlsbad, CA). Cells were co-transfected with miR-140 overexpressing plasmid. Luciferase activity was determined using the Dual-Luciferase assay system (Promega, Madison, WI) after 48 h of transfection. Luciferase activity was normalized to *Renilla* luciferase activity.

### Histopathology

Hematoxylin and Eosin (H&E) staining of lung tissue sections were performed following standard procedures to observe the morphology and the signs for fibrotic tissue. Lung tissue sections were also subjected to a modified Masson’s Trichrome staining (ScyTek Laboratories, West Logan, Utah) according to the manufacturer’s directions to determine the collagen deposition. Briefly, after the rehydration steps, sections were fixed with Bouin’s fluid, stained with Weigert’s hematoxylin, followed by incubation with Biebrich Scarlet-Acid Fuchsin, differentiation in a phosphomolybdic-phosphotungstic acid solution, incubation with Aniline Blue, and incubation with acetic acid. After dehydration, sections were mounted and visualized using a Nikon Eclipse Ti microscope (Nikon Instruments Inc.; Melville, NY).

### Immunofluorescence Staining and *In Situ* Hybridization of miR-140

Formalin fixed and paraffin-embedded sections were used for immunofluorescence staining as previously described^45^. After being permeabilized with PBS containing 0.2% Triton-X and blocked with PBS containing 10% Goat Serum and 1% BSA, the sections were incubated with primary antibodies overnight followed by fluorochrome–conjugated secondary antibodies (Life Technologies) and DAPI counterstaining. *In situ* hybridization for miR-140 was performed as previously described using 5′-digoxigenin-tagged probe^46^. Colorimetric detection reaction was performed using NBT/BCIP (Roche; Indianapolis, IN, USA) for 48 h. The images were captured using Nikon Eclipse Ti (Nikon Instruments Inc.; Melville, NY, USA). Antibodies against α-SMA, Smad3, fibronectin and F4/80 were purchased from Abcam (Cambridge, MA). Antibodies for CD197/Alexa Fluor 647, CD38/PE, CD206/Alexa Fluor 488 were purchased from Biolegend (San Diego, CA). The intensity of immunofluorescence was quantified using Image J software (NIH) as previously described^47^.

### Statistical Analysis

Statistical analysis was performed using the Graph Pad Prism software and data were assessed by the 2-tailed Student t test. A difference was considered significant when P < 0.05 (*) or P < 0.01 (**). Data are presented as mean ± S.D.

## Additional Information

**How to cite this article**: Duru, N. *et al*. Loss of miR-140 is a key risk factor for radiation-induced lung fibrosis through reprogramming fibroblasts and macrophages. *Sci. Rep.* **6**, 39572; doi: 10.1038/srep39572 (2016).

**Publisher's note:** Springer Nature remains neutral with regard to jurisdictional claims in published maps and institutional affiliations.

## Supplementary Material

Supplementary Information
