# Supplementary material for: Loss of miR-140 is a key risk factor for radiation-induced lung fibrosis through reprogramming fibroblasts and macrophages
Source: Sci Rep. 2016 Dec 20;6:39572. doi: 10.1038/srep39572 (PMC5172237; doi:10.1038/srep39572)
Supplement: Supplementary Information [file srep39572-s1.pdf]

Supplementary Information for

Loss of miR-140 is a key risk factor for radiation-induced lung fibrosis through reprogramming fibroblasts and macrophages

Nadire Duru, Yongshu Zhang, Ramkishore Gernapudi, Benjamin Wolfson, Pang-Kuo Lo, Yuan Yao, Qun Zhou

Department of Biochemistry and Molecular Biology, Greenebaum Cancer Center, University of Maryland School of Medicine, Baltimore, MD 21201, USA.

Corresponding Author:
Qun Zhou, M.D., Ph.D
Mailing Address: 108 N. Greene St., Biomedical Research Facility, R337, Baltimore, MD 21201
Phone: (410) 706-1615
Fax: (410) 706-8297
e-mail address: qzhou@som.umaryland.edu

This file include:

1. Supplementary Figure S1
2. Supplementary Figure S2
3. Supplementary Figure S3

SUPPLEMENTARY FIGURE LEGENDS

Supplementary Figure S1. Quantification of the immunofluorescent staining data. **A,** Fibrotic lung tissues shows the increased expression of α-SMA, Smad3 and fibronectin compared to non-fibrotic lung tissues. **B,** miR-140 knockout lung tissue has increased α-SMA, Smad3 and fibronectin expression compared to wild type lung tissue.

Supplementary Figure S2. Full-length blots of cropped images shown in Figure 3C for wild type MLFs with or without FIR treatment and miR-140 knockout MLFs with or without FIR treatment. **A,** Full-length blot showing the expression of fibronectin, α-SMA and β-actin. **B,** Full-length blot showing the expression of Smad3 and α-SMA. **C,** Full-length blot showing the expression of fibronectin and α-SMA.

Supplementary Figure S3. Full-length blots of cropped images shown in Figure 4E for L-929 control cells, L-929 cells transfected with miR-140 overexpressing plasmid, L-929 cells treated with 2ng/ml TGF-β and L-929 cells transfected with miR-140 overexpressing plasmid and treated with 2ng/ml TGF-β. **A,** Full-length blot showing the expression of α-SMA. **B,** Full-length blot showing the expression of Smad3 and vinculin. **C,** Full-length blot showing the expression of fibronectin. **D,** Full-length blot showing the expression of β-actin. **E,** Full-length blot showing the expression of vinculin.

S1A

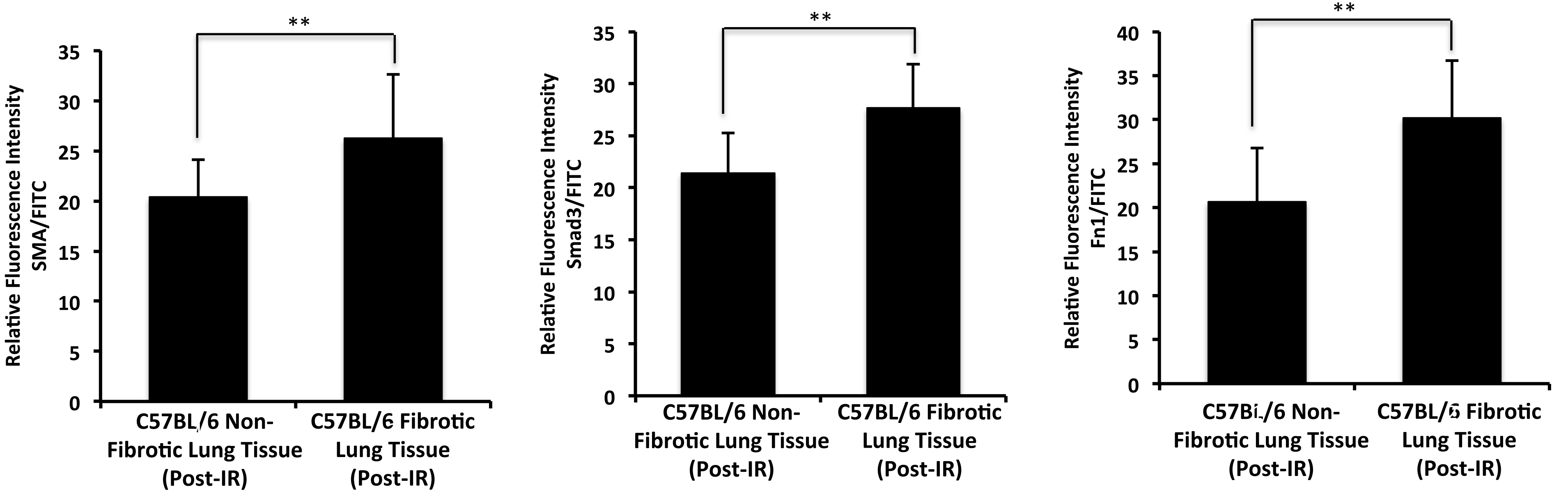
**
Relative Fluorescence Intensity
SMA/FITC
0
5
10
15
20
25
30
35
C57BL/6 Non-Fibrotic Lung Tissue (Post-IR)
C57BL/6 Fibrotic Lung Tissue (Post-IR)
**
Relative Fluorescence Intensity
Smad3/FITC
0
5
10
15
20
25
30
35
C57BL/6 Non-Fibrotic Lung Tissue (Post-IR)
C57BL/6 Fibrotic Lung Tissue (Post-IR)
**
Relative Fluorescence Intensity
Fn1/FITC
0
5
10
15
20
25
30
35
40
C57BL/6 Non-Fibrotic Lung Tissue (Post-IR)
C57BL/6 Fibrotic Lung Tissue (Post-IR)

S1B

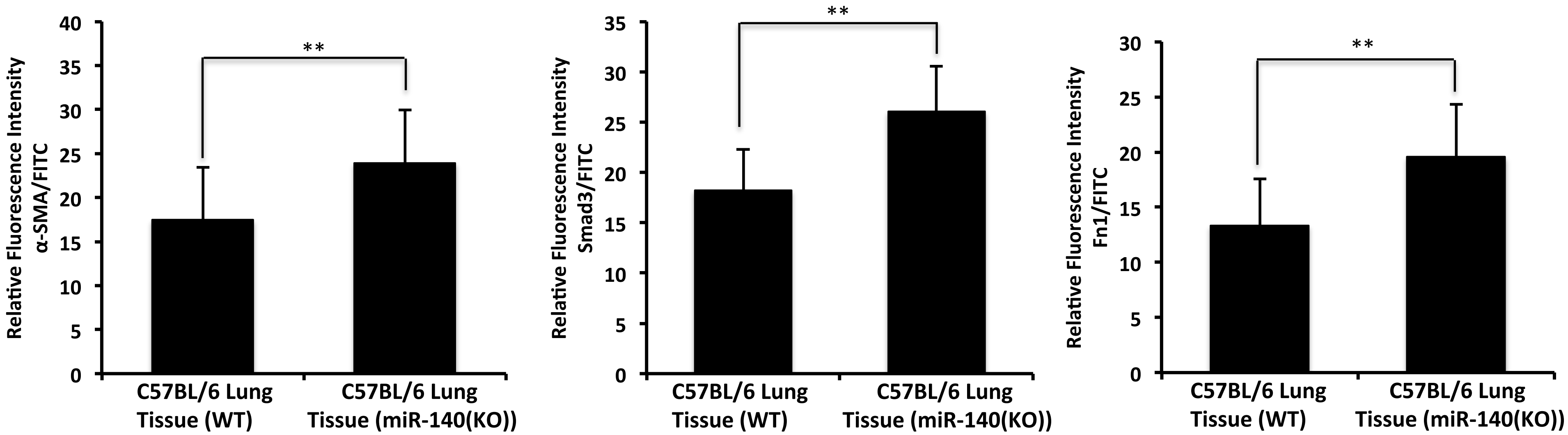
**
Relative Fluorescence Intensity
α-SMA/FITC
0
5
10
15
20
25
30
35
40
C57BL/6 Lung Tissue (WT)
C57BL/6 Lung Tissue (miR-140(KO))
**
Relative Fluorescence Intensity
Smad3/FITC
0
5
10
15
20
25
30
35
C57BL/6 Lung Tissue (WT)
C57BL/6 Lung Tissue (miR-140(KO))
**
Relative Fluorescence Intensity
Fn1/FITC
0
5
10
15
20
25
30
C57BL/6 Lung Tissue (WT)
C57BL/6 Lung Tissue (miR-140(KO))

S2A

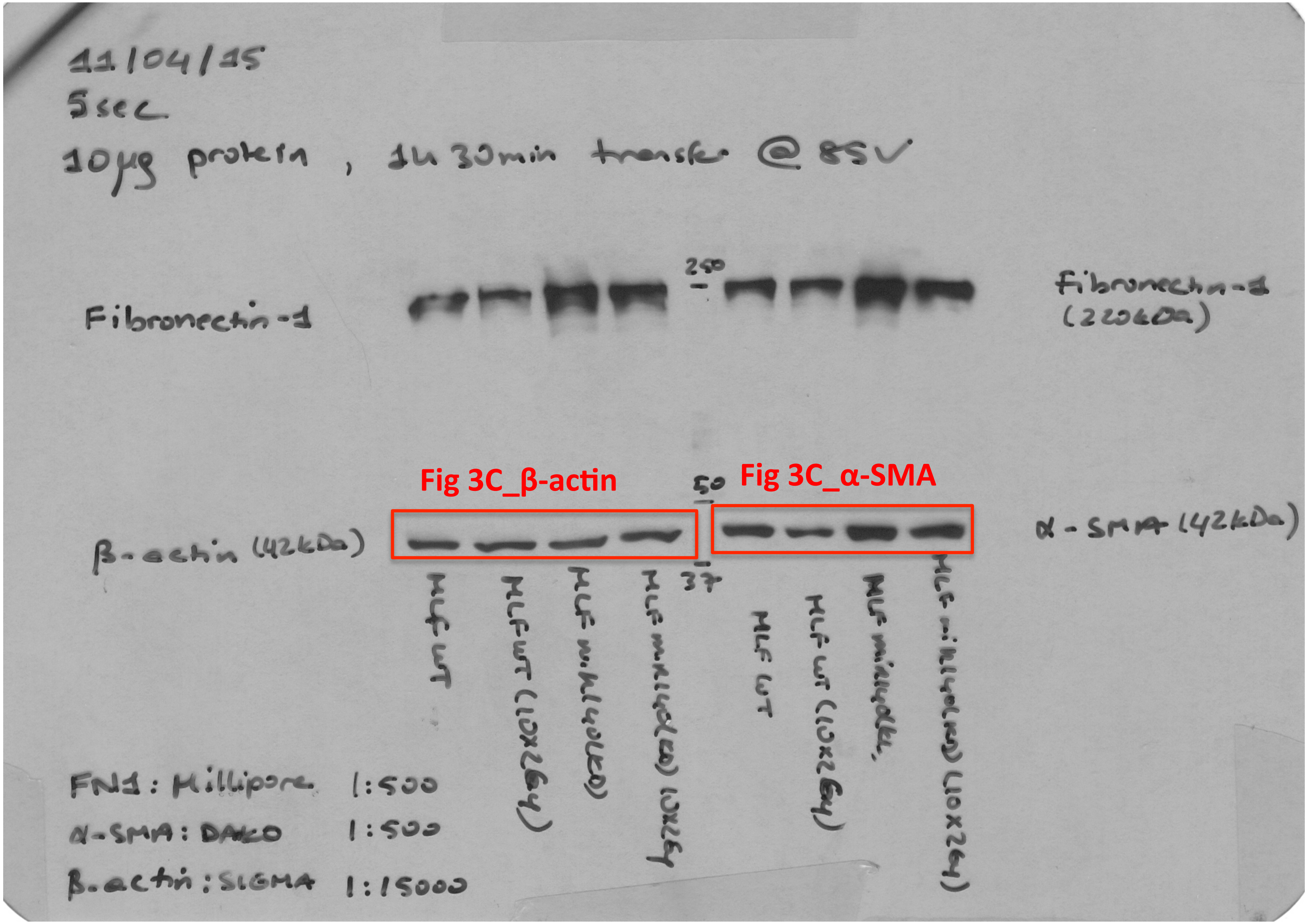
11/04/15
5sec
10µg protein, 1h 30min transfer @ 85V
Fibronectin-1
250
Fibronectin-1 (220kDa)
Fig 3C_β-actin
Fig 3C_α-SMA
50
β-actin (42kDa)
α-SMA (42kDa)
37
MLF WT
MLF WT (10x2Gy)
MLF WT
MLF WT (10x2Gy)
FN1: Millipore 1:500
α-SMA: DAKO 1:500
β-actin: SIGMA 1:15000

S2B

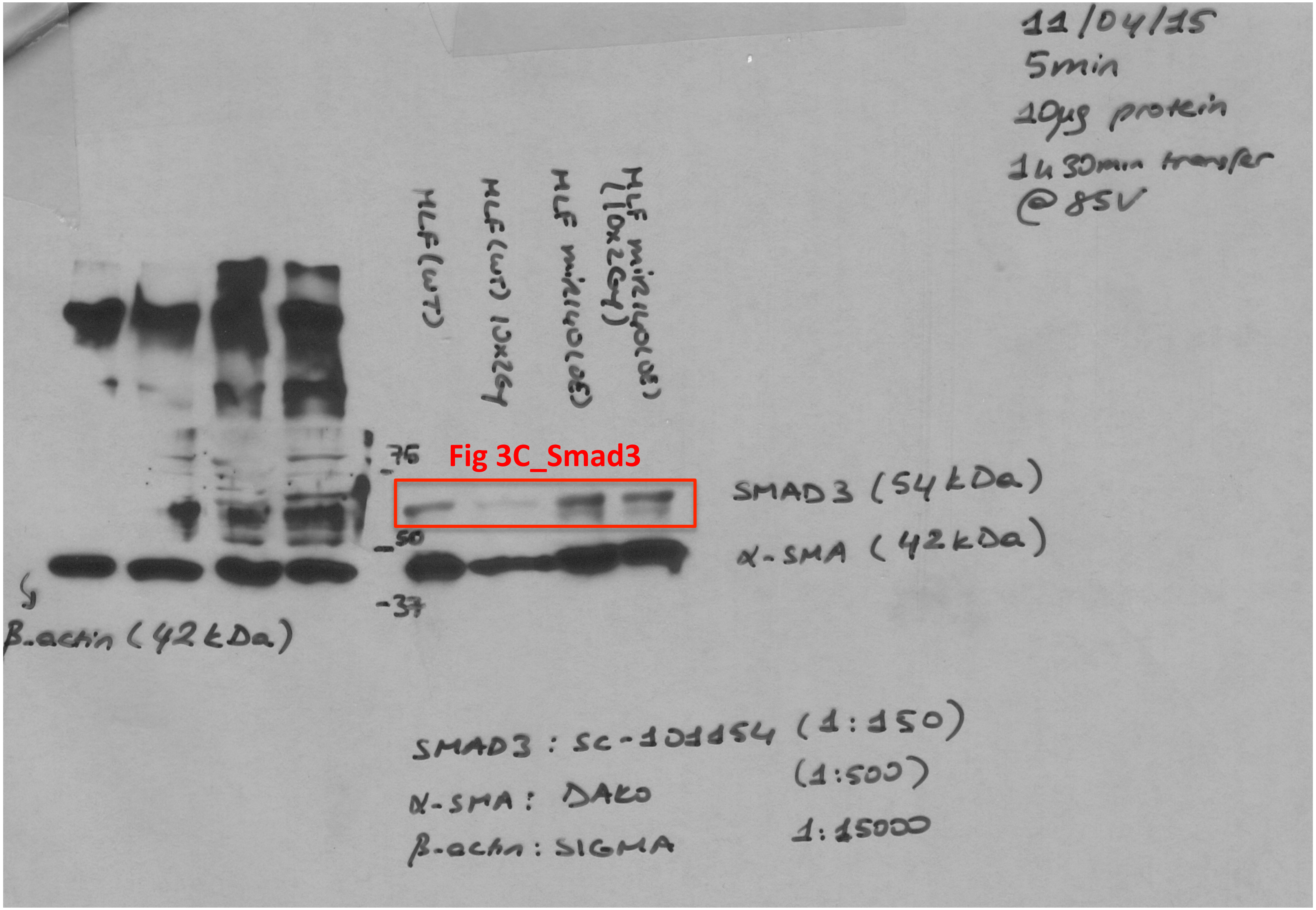
11/04/15
5min
10µg protein
1h 30min transfer
@85V
MLF (WT)
MLF (WT) 10x2Gy
MLF miR140(OE)
MLF miR140(OE) (10x2Gy)
75
Fig 3C_Smad3
SMAD3 (54kDa)
50
α-SMA (42kDa)
37
β-actin (42kDa)
SMAD3 : sc-101154 (1:150)
α-SMA : DAKO (1:500)
β-actin : SIGMA 1:15000

S2C

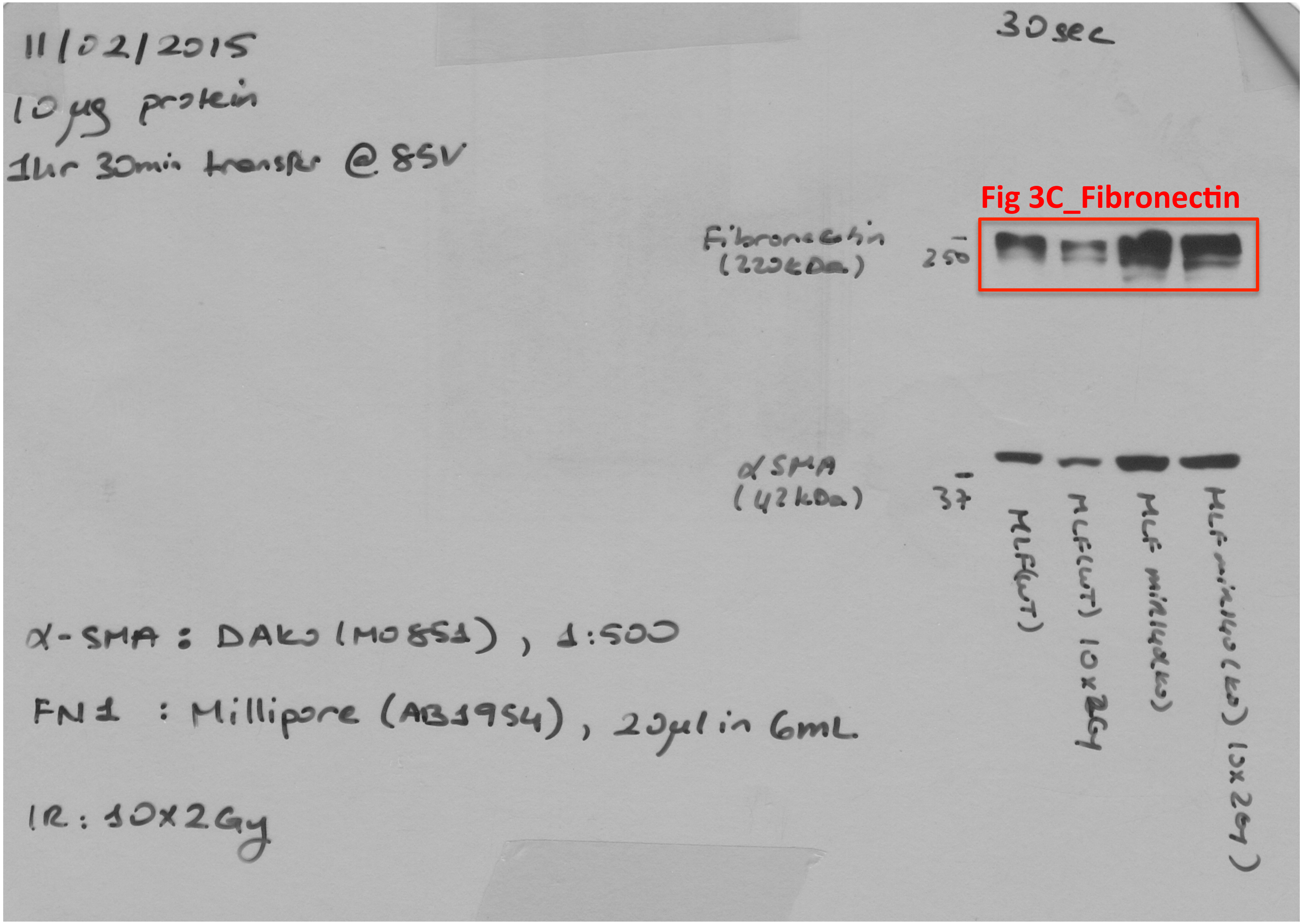
11/02/2015
10 µg protein
1hr 30min transfer @ 85V
30 sec
Fig 3C_Fibronectin
Fibronectin (220kDa)
250
αSMA (42kDa)
37
MLF(WT)
MLF(WT) 10x2Gy
MLF mir140(ko)
MLF mir140(ko) (10x2Gy)
α-SMA : DAKO (M0851), 1:500
FN1 : Millipore (AB1954), 20µl in 6mL
IR: 10x2Gy

S3A

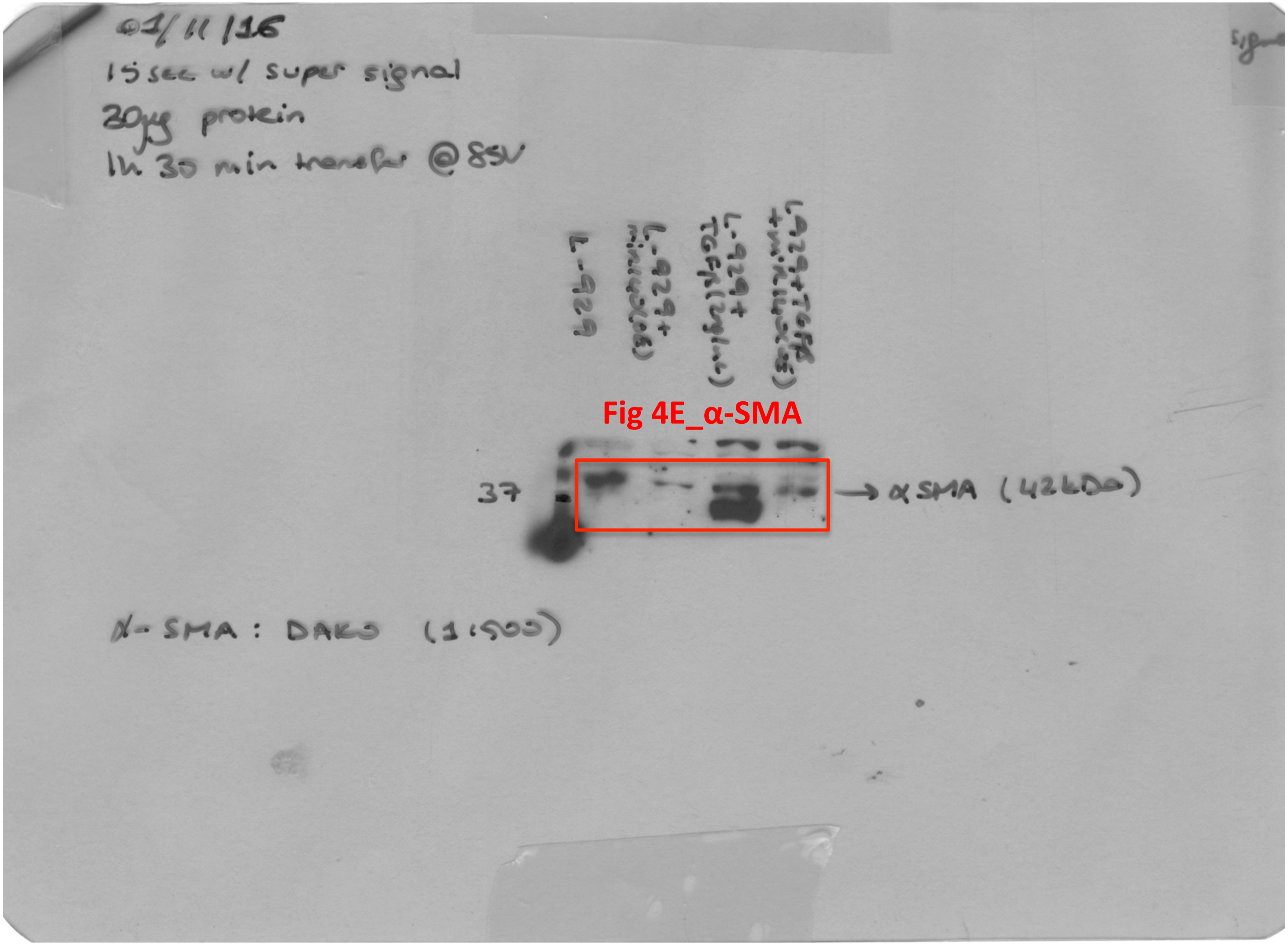
01/11/16
15 sec w/ super signal
30μg protein
1h 30 min transfer @ 85V
Fig 4E_α-SMA
37
→ α SMA (42kDa)
α-SMA : DAKO (1:500)

S3B

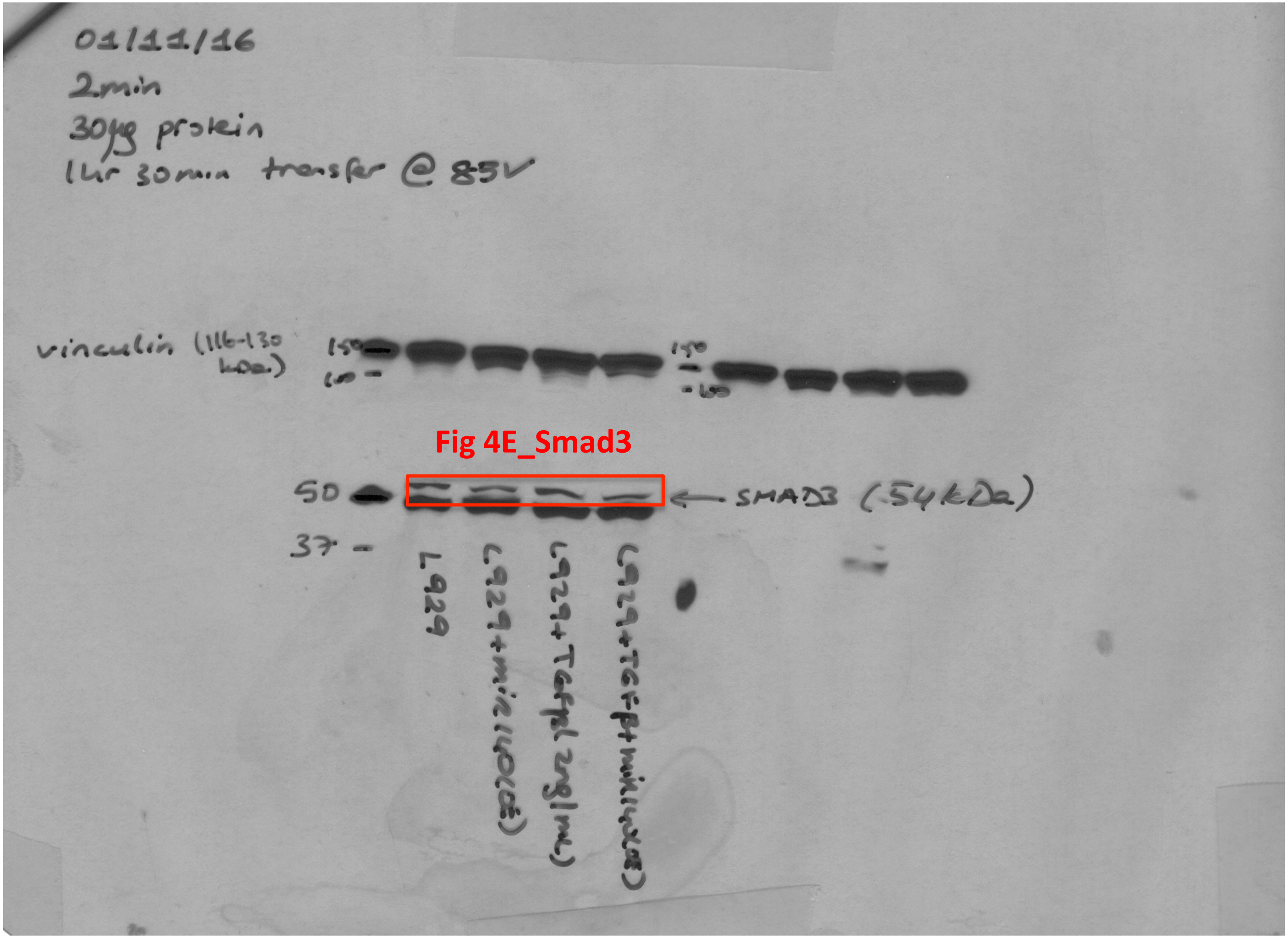
01/11/16
2min
30µg protein
1hr 30min transfer @ 85V
vinculin (116-130 kDa)
150
100
Fig 4E_Smad3
50
37
SMAD3 (54kDa)
L929
L929+mir140(OE)
L929+TGFβ1 2ng/mL)
L929+TGFβ+mir140(OE)

S3C

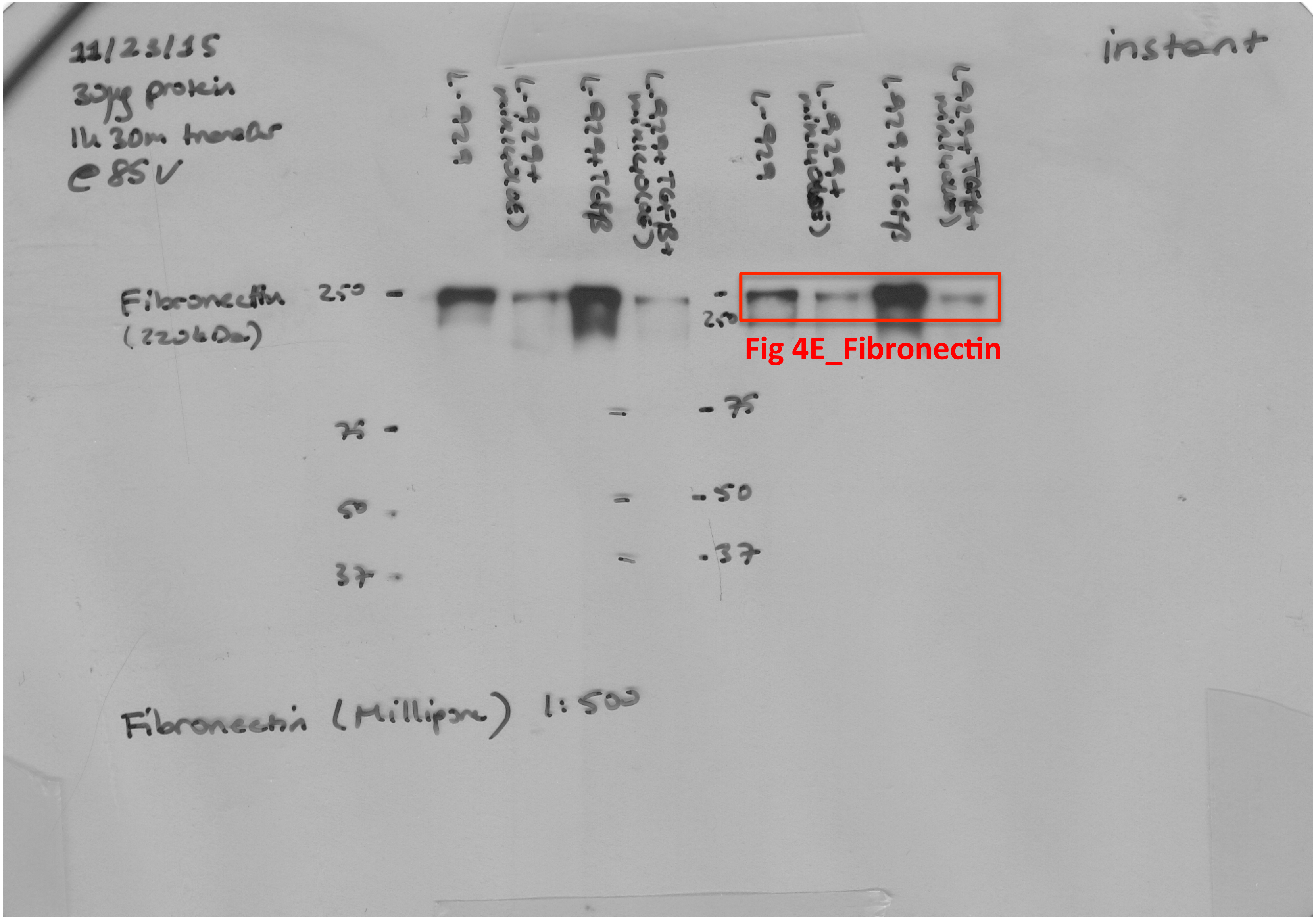
11/23/15
30μg protein
1h 30m transfer
@ 85V
instant
Fibronectin
(220kDa)
250
75
50
37
Fig 4E_Fibronectin
Fibronectin (Millipore) 1:500

S3D

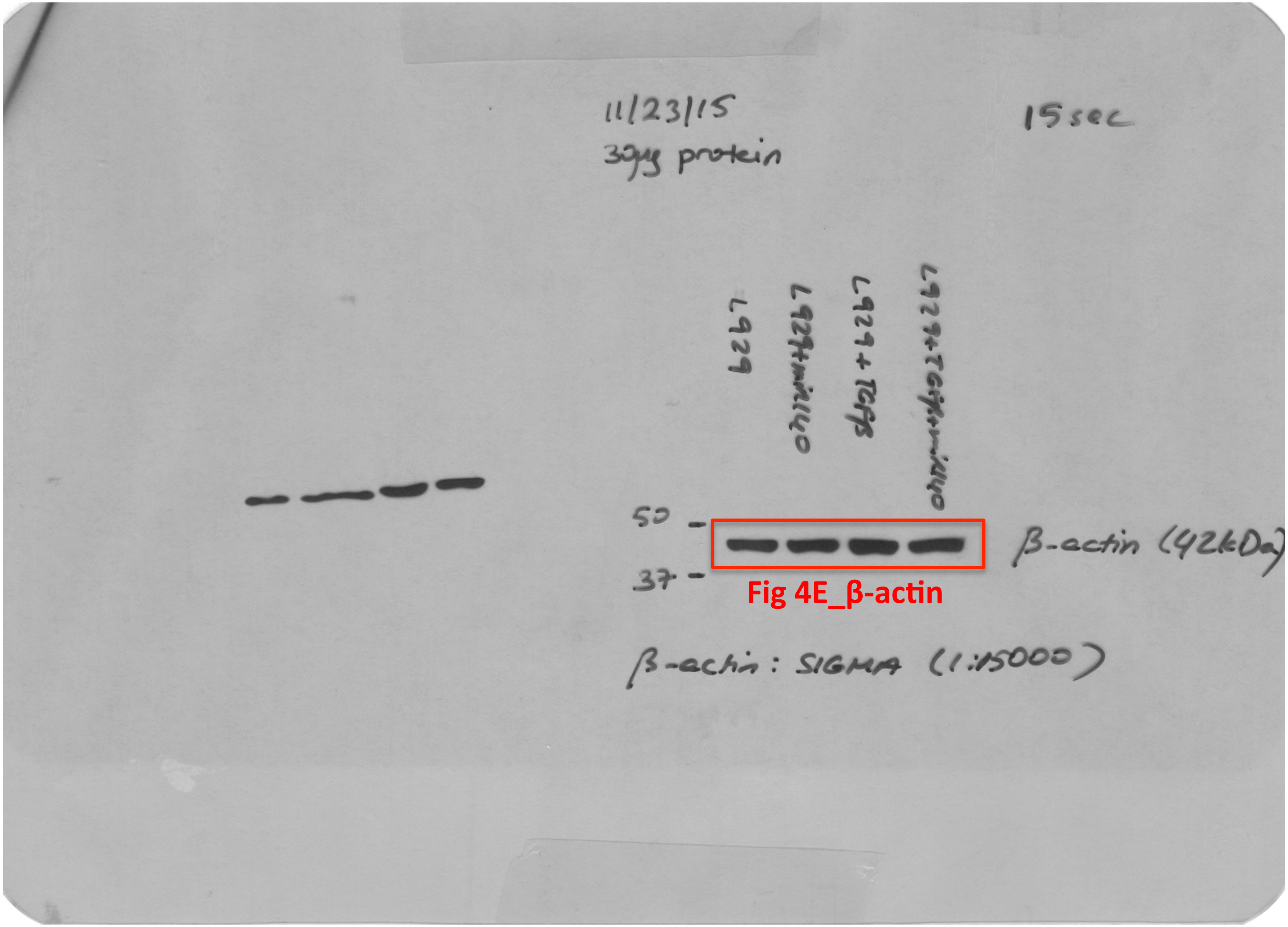
11/23/15
30µg protein
15 sec
L929
L929+mir140
L929+TGFβ
L929+TGFβ+mir140
50
37
β-actin (42kDa)
Fig 4E_β-actin
β-actin : SIGMA (1:15000)

S3E

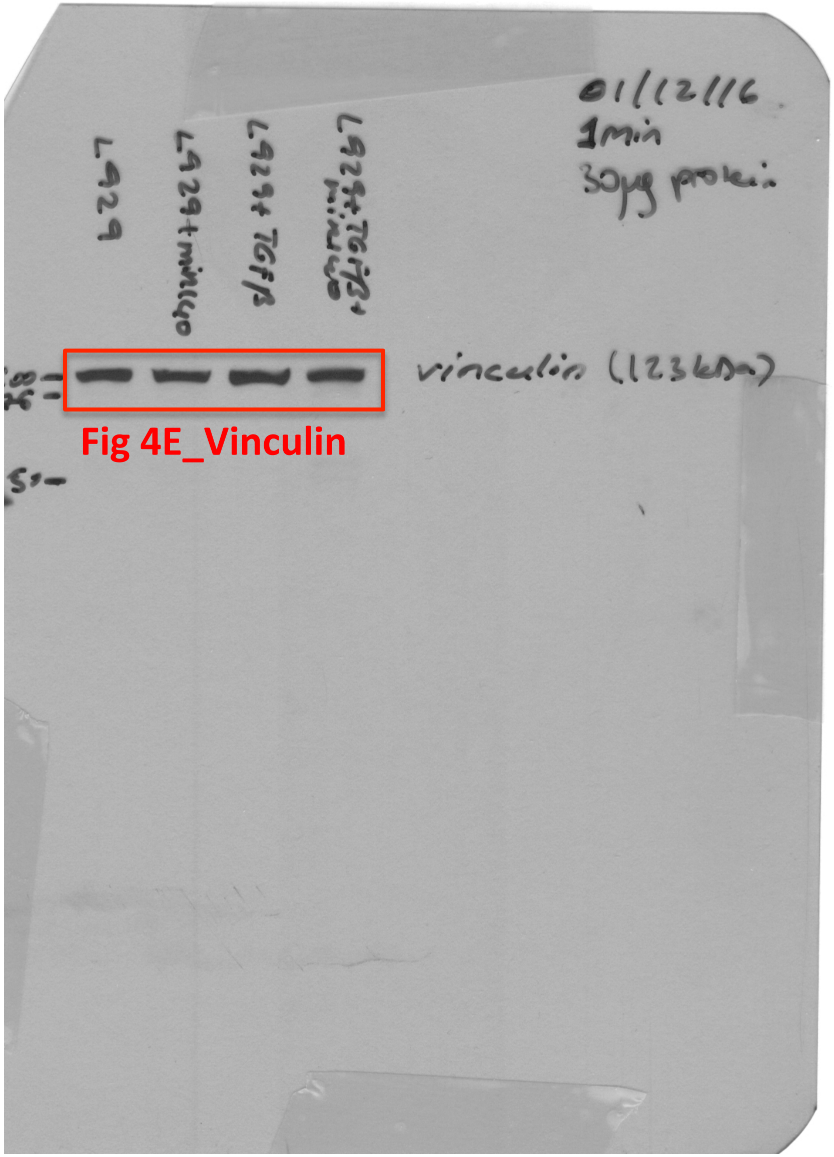
01/12/16
1min
30μg protein
L929
L929+TGFβ
vinculin (123kDa)
Fig 4E_Vinculin
